# Smoothness of Gait in Overweight (But Not Obese) Children Aged 6–10

**DOI:** 10.3390/bioengineering10030286

**Published:** 2023-02-22

**Authors:** Micaela Porta, Demetra Cimmino, Bruno Leban, Federico Arippa, Giulia Casu, Maria Chiara Fastame, Massimiliano Pau

**Affiliations:** 1 Department of Mechanical, Chemical and Materials Engineering, University of Cagliari, Piazza d’Armi, 09123 Cagliari, Italy; 2 Department of Pedagogy, Psychology, Philosophy, University of Cagliari, Piazza d’Armi, 09123 Cagliari, Italy

**Keywords:** gait, overweight, children, harmonic ratio (HR), symmetry, smoothness

## Abstract

Excessive body mass represents a serious threat to the optimal psychophysical development of children, and it is known to be able to significantly affect their locomotor capabilities, making them more prone to the development of musculoskeletal disorders. However, despite the relevant number of existing studies, a clear gait pattern of overweight children has not been defined yet, particularly in the case of a mass excess that is relatively small (i.e., in those not obese). In the present study, we employed a wearable inertial measurement unit placed on the low back to derive spatio-temporal parameters and quantify the smoothness of gait (by means of harmonic ratio) from trunk accelerations acquired during gait trials carried out by 108 children aged 6–10 (46% males), stratified into two groups according to their body mass index (normal weight, *n* = 69 and overweight, *n* = 39). The results show that while gait speed, stride length, cadence and double support duration were found to be almost identical in the two groups, significant differences were observed in terms of harmonic ratio. In particular, overweight children exhibited a reduced harmonic ratio in the antero-posterior direction and higher harmonic ratio in the medio-lateral direction. While the significantly lower harmonic ratio in the antero-posterior direction is likely to be indicative of a loss of smoothness in the walking direction, probably due to a combination of factors associated with the altered movement biomechanics, the higher harmonic ratio in the medio-lateral direction might be associated with specific strategies adopted to increase lateral stability. Although further studies are necessary to elucidate the specific mechanisms that influence the smoothness of gait, it is noteworthy that harmonic ratios appear sensitive even to subtle change in locomotor control in overweight children characterized by apparently regular spatio-temporal parameters of gait and might be employed to assess the effectiveness of interventions designed to improve mobility functions.

## 1. Introduction

Worldwide epidemiological data report that, over the last four decades, childhood obesity appears characterized by an increasing trend of growth, although with some differences depending on the geographical area. For instance, in most high-income industrialized countries, where such a phenomenon came forward earlier, in the last few years, the prevalence of overweight and obesity, although high, has reached a sort of plateau, while it continues to steadily increase in low-income and middle-income countries [1,2].

Among the wide spectrum of medical comorbidities (which involve both physical and psychological dimensions) associated with a mass excess, a commonly encountered issue is represented by the relevant changes in biomechanical functions that occur during the execution of both simple and complex motor tasks. Such alterations, in combination with the increased risk of musculoskeletal problems (i.e., malalignment, pain, osteoarthritis, etc.), ultimately lead to a reduction in mobility, physical activity and overall quality of life [3,4]. In particular, a significant part of the research on mobility issues in overweight children attempted to quantitatively characterize the possible alterations of gait patterns associated with mass excess by analyzing several kinematic and kinetic parameters. Despite the significant heterogeneity of the studies carried out in the last few decades in terms of age range, male-to-female ratio, percentage of overweight and obese participants and experimental setup, they suggest that there is strong evidence as regards the existence of significant kinematic alterations and differences in generated/absorbed power at the hip/pelvis and knee joints (see the review of Molina-Garcia et al. for details [5]). In contrast, the level of evidence is inconsistent for most spatio-temporal parameters and only moderate in the case of step width and stance phase duration (which have been found to be significantly increased due to mass excess). Such findings have two important consequences, namely (1) the most important differences in gait biomechanics (kinematics and kinetics) can be detected only using specialized laboratory equipment, including motion capture systems and force platforms, and (2) spatio-temporal parameters of gait appear substantially unsuitable to identify differences associated with mass excess (except for step width and stance phase duration, as previously mentioned), especially when of small magnitude.

In this context, particularly appealing is the possibility to employ wearable inertial sensors (i.e., inertial measurement units, IMUs) to obtain information about gait patterns based on trunk/limb acceleration data. Such devices gained popularity among the researchers involved in the quantitative assessment of human movement [6,7] due to their miniaturized size, easiness of use, affordable cost and, above all, because they allow the overcoming of several limitations typical of motion capture systems, such as the need to have available a dedicated space, to perform specific preparations for the tested subjects (i.e., undressing, anthropometric collection data, marker placement, etc.) and the overall conditions in which the test takes place, which make this type of analysis uneconomical. In particular, the configuration, which consists of a unique sensor (usually located on the low back, close to the body’s center of mass), is characterized by minimal encumbrance for the individual, fast setup and the possibility to perform tests in a variety of settings (including clinics, schools, gyms, outdoor settings), terrain conditions, overground or treadmill walking, straight or curved paths, etc. While such an approach is commonly employed to obtain the spatio-temporal parameters of gait (by processing the acceleration data with suitable algorithms [8]), an interesting feature associated with the use of IMUs when they are located on the low back is the possibility to characterize different aspects of gait using metrics directly derived from trunk acceleration data. In this regard, several different approaches in terms of acceleration processing and the calculation of associated metrics, which cover important characteristics of the motor control underlying the realization of gait patterns, such as smoothness, efficiency, automaticity, adaptability, variability, stability and symmetry, have been proposed [9]. For the purposes of the present study, we will focus our attention on the smoothness of gait, which is a feature able to summarize the consistent forward progression and the repeatability of step patterns (i.e., step-to-step symmetry).

The use of trunk accelerations to detect and characterize the existence of possible alterations of gait dates back to the 1970s, when Smidt et al. [10] proposed an approach based on the harmonic analysis of the three orthogonal accelerations recorded at the low-back level in the frequency domain. In particular, they suggested that the value of a parameter called the harmonic ratio (HR), obtained as the ratio of the sum of the coefficients of the even harmonics and the sum of the coefficients of the odd harmonics, could represent a measure of the smoothness of gait and demonstrated that several types of gait alterations (associated either with orthopedic and neurologic conditions or even with the use of walking aids) were able to affect smoothness by reducing the HR values. In the last two decades, even exploiting the remarkable advancements of the micro-electro-mechanical systems (MEMS) technology, such concepts have been refreshed, enriched and applied to a variety of studies on gait involving both general and clinical populations. It is now generally recognized that HRs represent a reliable marker of whole-body balance during gait [11,12] and are able to discriminate changes in the smoothness of gait associated with gait maturation [13,14,15,16] and aging [12,17]. Moreover, it has been demonstrated that HR values are altered in the presence of neurologic and orthopedic conditions [18,19,20], even when spatio-temporal parameters appear physiological [21] and in older adults with cognitive deficits [22]. Lastly, HRs can be used, in combination with other parameters, to assess either the effectiveness of rehabilitative treatments targeted at gait function [23,24] or the outcome of orthopedic surgery that involves the lower limbs [25,26].

It is noteworthy that a limited number of studies investigated the possible changes occurring in the smoothness of gait due to body mass abnormalities (i.e., thinness or overweight/obesity) and, to our knowledge, only one specifically tested children. Misu et al. [27] reported that, among community-dwelling older adults, malnutrition is associated with reduced values of HR in the medio-lateral direction, hypothesizing that nutritional status might play a relevant role in the lateral trunk control during walking. Cimolin et al. [28] analyzed the smoothness of gait in 75 children aged 7–14, stratified into three groups composed, respectively, of underweight, normal-weight and overweight/obese participants. They found that while the spatio-temporal parameters of gait did not differ significantly across groups, the HR in the medio-lateral direction significantly increased while passing from under- to overweight and the HR in the antero-posterior and superior-inferior directions was significantly lower in those underweight with respect to overweight. Moreover, HRs in all directions were found to be significantly correlated with body mass index (BMI). Lastly, significantly reduced values of HR in the antero-posterior and superior-inferior directions were found in young adults affected by Prader–Willi syndrome, a genetic developmental disability that represents the most commonly known genetic cause of obesity [29].

In summary, although the analysis of gait smoothness seems to represent a promising source of information to support the accurate and detailed quantitative characterization of gait patterns in individuals with body mass alterations, the available data are scarce and heterogenous. To partly overcome such limitations, in the present study, we aimed to assess the smoothness of gait in a sample of overweight (but not obese) primary schoolchildren, to test the hypothesis that, even they still have not reached the obesity condition and the spatio-temporal parameters of their gait are weakly (or not at all) changed in comparison with those of normal-weight peers, some signs of gait alteration are detectable in terms of HR values.

## 2. Materials and Methods

### 2.1. Participants

In the period March–May 2022, the Laboratory of Biomechanics and Industrial Ergonomics of the University of Cagliari (Italy), in collaboration with two primary schools of the province of Cagliari (Italy), launched a call for a screening of balance, gait and functional mobility in schoolchildren aged 6–10. Of the 284 children who regularly attended the schools, 224 (79%) agreed to participate as they and their parents signed an informed consent form that included a detailed explanation of the purposes of the study and of the testing procedure. Although no inclusion or exclusion criteria were explicitly defined in the call, the parents of the schoolchildren were asked to report the existence of either musculoskeletal or neurologic conditions able to significantly impair balance and walking abilities. In this regard, although it was planned to test all children who agreed to participate (this was also the intention of the school, with the specific purpose to avoid the possibility that some children felt “different” or “excluded”), we took care not to consider for the study individuals with prosthetic or orthotic devices or any other birth defect able to influence gait and balance. The study, which was carried out according to the principles expressed in the Declaration of Helsinki and its later amendments, was approved by the Ethics Committee of the University of Cagliari (authorization number 2022-UNCACLE-0001470).

Experimental tests were performed directly at participating schools during regular days of lessons, in dedicated spaces made available by the School Board. Children were called in small groups and, preliminarily, their height and body mass were recorded using an ultrasonic digital height meter (Soehnle 5003, Soehnle, Backnang, Germany) and a digital scale (RE310, Wunder, Milan, MI, Italy). The subsequent calculation of BMI (obtained by the ratio body mass/height^2^) allowed us to classify them as normal-weight, overweight or obese according to the cut-off points defined by Cole et al. [30]. Although all the participants were tested, for the purposes of the present study, the attention was focused on the group composed of overweight children (*n* = 39, 17.4% of the whole cohort) and on a control group of 69 normal-weight children matched for age, sex and height. Obese children (*n* = 10, 4.5% of the whole cohort) were not included in the analysis. The demographic and anthropometric features of the children selected for the analysis are reported in Table 1.

### 2.2. Gait Data Acquisition

Instrumental gait analysis was performed using a commercially available, wearable inertial sensor (G-Sensor^®^, BTS Bioengineering, Milan, MI, Italy), which was previously employed in similar studies involving children and adolescents [16,31,32,33]. The device was inserted into the pocket of a semi-elastic belt attached to the participant’s waist in such a way as to have it positioned approximately at the L4-L5 vertebrae level. Children were then instructed to stand still for few seconds (to ensure the proper calibration of the sensor) and, following a verbal signal, to walk down a 35-m hallway along a straight trajectory at a self-selected speed and in the most natural way possible. Such distance approximately corresponded to a number of strides in the range of 20–40 depending on the child’s stature. The sensor acquired the trunk accelerations along three orthogonal axes, namely antero-posterior (AP, which corresponds to the walking direction), medio-lateral (ML) and supero-inferior (SI), at 100 Hz frequency and transmitted them in real time via Bluetooth to a PC. Such data were then post-processed by means of a custom Matlab^®^ routine that discarded the first and last two strides (to exclude the effects of acceleration and deceleration transients) and we calculated the following variables of interest:

- Spatio-temporal parameters of gait (namely gait speed, stride length, cadence and duration of double support phase expressed as a percentage of the gait cycle). The parameters known to be influenced by an individual’s anthropometry (i.e., gait speed, stride length and cadence) were normalized by dividing them by each participant’s height [34,35,36].
- HRs for AP, ML and SI directions.

Spatio-temporal parameters of gait were calculated using the peak detection algorithm proposed by Zijlstra [37], which essentially consists of processing the acceleration components (signals) in order to identify the characteristic patterns of trunk movement during gait as predicted by the inverted pendulum model. Instead, the calculation of the HRs was carried out following the approach proposed by Pasciuto et al. [38]. In short, the accelerations acquired during the gait trials were preliminarily processed in the frequency domain using a finite Fourier series, and then the HRs were calculated by dividing the square of the amplitude of the first ten even harmonics (AP and SI directions) or odd harmonics (ML direction) by the sum of the squares of the amplitudes of the first twenty even and odd harmonics. Such a ratio was then multiplied by 100, thus providing an index of easy interpretation that may assume values from 0 (in case of total asymmetry) to 100 (perfect symmetry). Previous studies indicated that, in case of healthy children and adolescents, the values range from 89 to 95 (AP and SI direction) and from 81 to 86 (ML direction) [16].

### 2.3. Statistical Analysis

Preliminarily, parametric model assumptions were verified for all variables of interest (i.e., normality, homogeneity and presence of outliers). Thus, the existence of possible differences introduced in the spatio-temporal parameters of gait by mass excess was assessed using a one-way multivariate analysis of variance (MANOVA). The independent variable was the group (i.e., normal or overweight) and the dependent variables were the 4 spatio-temporal parameters previously listed. As regards the HRs, considering that previous studies reported a significant influence of gait speed on their values (i.e., HRs tend to increase with increasing speed [12]), a one-way multivariate analysis of covariance (MANCOVA) was performed, including gait speed as a covariate. In all cases, the level of significance was set at *p* = 0.05. Univariate ANOVAs were carried out as a post-hoc test by reducing the level of significance to *p* = 0.0125 (0.05/4) for spatio-temporal parameters and *p* = 0.016 (0.05/3) for HRs after a Bonferroni correction for multiple comparisons. All analyses were carried out using the IBM SPSS Statistics v.23 software (IBM, Armonk, New York, NY, USA).

## 3. Results

Spatio-temporal parameters of gait and HRs for the two groups of children are reported in Table 2.

The statistical analysis did not detect a significant main effect of mass excess on the spatio-temporal parameters of gait, either when considering the absolute values [F(4,103) = 0.78, *p* = 0.533, Wilks λ = 0.97, η^2^ = 0.03] or the normalized values [F(4,103) = 0.88, *p* = 0.46, Wilks λ = 0.98, η^2^ = 0.017].

**Figure 1:**
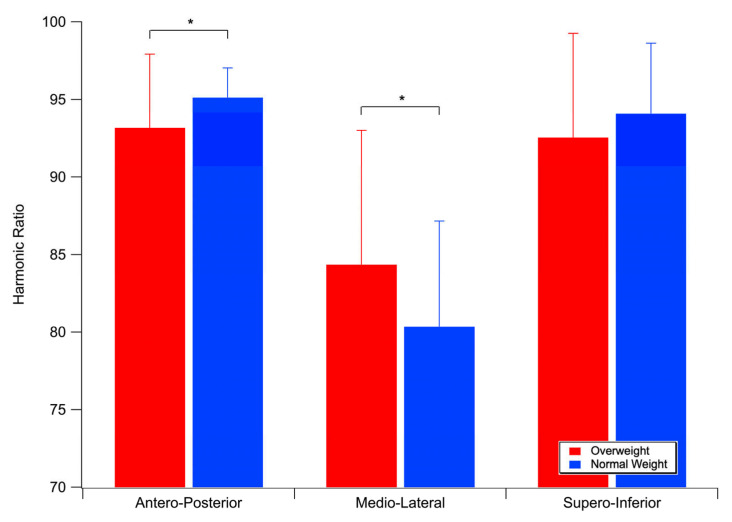
Trend of HR for normal-weight and overweight children. Values are displayed as mean and SD. The symbol * denotes a significant difference vs. normal-weight children after Bonferroni correction (*p* = 0.016).

As regards the smoothness of gait parameters (see Figure 1), after controlling for gait speed, the MANCOVA detected a significant main effect of overweight on HR values [F(3,103) = 12.22, *p* < 0.001, Wilks λ = 0.74, η^2^ = 0.26]. In particular, the post-hoc analysis revealed that, compared with their normal-weight peers, overweight children were characterized by significantly lower HR values in the AP direction (93.18 vs. 95.13, *p* = 0.003) and higher HR values in the ML direction (84.36 vs. 80.35, *p* = 0.008). No significant differences were found between groups for HRs in the SI direction.

## 4. Discussion

The present work aimed to assess the existence of possible alterations in the main spatio-temporal parameters and in the smoothness of gait in a sample of primary schoolchildren aged 6–10 characterized by a mass excess that leads to classifying them as overweight but not obese. At first, our results demonstrated that, even though our sample of overweight participants was characterized by a BMI more than 30% higher than that of their normal-weight peers, the spatio-temporal parameters of gait were substantially similar across the two groups. As previously mentioned, there is no clear agreement in the existing literature about the actual influence of mass excess on such parameters; however, it should be noted that the recent review by Molina-Garcia et al. [5] pointed out that only the step width and stance phase duration are consistently altered in overweight and obese children (level of evidence: moderate). Although obtained from a sample relatively limited in size, our findings seem to suggest that changes in the main spatio-temporal parameters (i.e., speed, stride/step length and cadence) are expectable only in the case of obese children.

In contrast, significant differences emerged from the analysis of the HR, a parameter that has been interpreted as indicative of smoothness, rhythmicity and dynamic stability and that has been demonstrated to be effective in detecting subtle alterations in locomotor mechanisms that are not expressed through significant variations in spatio-temporal parameters [11]. Indeed, our results show that overweight children are characterized by worse smoothness in AP (i.e., the walking direction), as indicated by the reduced HR, but, at the same time, they exhibit higher HR values in ML. What explanation can be provided for such apparently contradictory results? At first, it should be recalled that, as regards the AP direction, the data of previous studies indicate no significant differences between normal weight and overweight/obese [28] or, consistent with the results of the present study, significantly lower values were observed in those who were obese [29]. Even though the overall amount of data is limited, we can hypothesize that such a lack of agreement could be attributed to differences in the sample compositions in terms of age and sex. In order to avoid any confounding effect, in the present study, we tried to carefully match any overweight participant with a normal-weight peer having very similar anthropometric and demographic features. Previous studies reported reduced smoothness in the AP direction in healthy older adults due to aging [17] and in the presence of neurologic diseases, even at early stages [19,21]. Interestingly, such conditions share the existence of impairments in postural control due to reduced performance of the sensory input system (i.e., vestibular, visual and proprioceptive), as well as to issues associated with their integration at a central level. It is, thus, possible that in overweight children, the alteration of postural control [39,40,41], which is likely due to the impaired effectiveness of the proprioceptive input originating from stress concentrations at the plantar region [42], possibly in combination with other factors able to affect the development of the lower limb musculoskeletal system (reductions in femoral anteversion, relative strength reductions, etc. [43]), can influence the whole-body movement during gait and, thus, consequently, reduce smoothness.

On the other hand, it was somewhat surprising to find that overweight children exhibit higher values of HR in the ML direction. In fact, such a result (which was previously observed even in a sample of children and adolescents who were overweight and obese by [28]) according to the commonly accepted interpretation of HR would suggest that they are characterized by better smoothness with respect to normal-weight individuals. However, there are several aspects that should be considered in order to correctly interpret such a finding. At first, although direct evidence is not available as the single-IMU configuration is unable to provide such data, it is possible that the gait pattern of our overweight children was characterized by a larger step width, a phenomenon that was previously reported in several studies [39,44,45,46]. The possible influence of step width on HR in ML was hypothesized by Raw-Lazzarini et al. [47], who found improved ML smoothness in older adults when walking on a treadmill with respect to an overground test; they attributed such changes to the different foot positions that characterized the two conditions [48]. On the other hand, Lowry et al. [49], who tested older adults walking at different speeds while adopting a narrow and wide base of support, did not find any difference in HR-ML in the case of preferred or wide step width. However, such a lack of difference could have been due to the fact that, in this latter case, the foot position was artificially constrained and not unconsciously adopted. Another interesting insight that could partly explain the ML results refers to the possibility that changes in step-to-step (AP and SI) and stride-to-stride (ML) smoothness characterize different aspects of gait, namely impairment or adaptability. To better clarify this hypothesis, we suggest that readers refer to the interesting papers of Moe-Nilssen et al. [50,51] and Helbostad et al. [52], who reported a similar apparent contradiction when examining the trunk acceleration variability. In particular, in the experiments described in [51], trunk acceleration variability during gait was calculated for two groups of older adults (fit and frail). The results showed that, as expected, frail subjects exhibited significantly higher variability in trunk accelerations in the AP and SI directions but, surprisingly, reduced variability in the ML direction. Helbostad et al. [52] found that older adults who underwent a fatigue protocol exhibited trunk acceleration variability during gait in the AP and SI directions that was higher with respect to non-fatigued individuals, but variability decreased in the ML direction. Such results have been interpreted by suggesting that not all features of the trunk acceleration can be considered representative of a situation of impairment. In particular, Moe-Nilssen suggested that different measures of variability may represent different aspects of locomotor control, being, for instance, those in the AP and SI directions indicative of “impairment”, and those of the ML direction indicative of “adaptability” [50]. Thus, we can speculate that the different alterations of HR in children, which originated from a mass excess, express different aspects of locomotor control and, thus, while HR in the AP direction can be considered a suitable measure of gait impairment, HR in the ML direction should be rather treated as an indicator of adaptability to the biomechanical and neuromuscular conditions imposed by overweight, which possibly reflect in changes in gait strategy (for instance, the increased step width).

Of course, the study has some limitations that should be acknowledged. Firstly, due to the relatively limited sample size, it was not possible to stratify the participants in such a way as to consider the effects of age and sex that might exist because of different trajectories of psychophysical development. Secondly, since the experimental trials were performed only in the case of level walking along a straight path at a self-selected speed, it has not been possible to investigate how other environmental conditions (e.g., curved paths, even and uneven terrains) or walking speeds influence HR values [12,17,18]. Moreover, since we did not have available a detailed medical record for the participants, nor did we have the chance to directly perform an accurate screening for the presence of pathological lower-extremity abnormalities (for instance, pes planus, genu varum or valgum, etc.), we are not able to clarify how the possible presence of such conditions could have affected the results. Lastly, as previously mentioned, it is possible that specific features of the body composition of overweight children might be responsible for some of the observed alterations in trunk acceleration (and, thus, consequently in HR values). In particular, further studies should be carried out in the future to elucidate the mechanisms by which factors such as fat distribution across the body, lower-extremity or appendicular lean mass, muscle mass of the lower extremities, etc., are able to influence the smoothness of gait.

## 5. Conclusions

Overweight (but not obese) children exhibit substantially similar gait patterns in terms of spatio-temporal parameters. However, the analysis of the gait smoothness, quantified by means of HR values, detected significant differences, with respect to normal-weight children, which involve both the AP and ML directions. While few data are available for individuals characterized by mass excess, the existing literature suggests that a reduction in HR in the AP direction is indicative of worse smoothness of gait and, thus, in this context, the use of HR would allow the detection of subtle alterations of gait even in the presence of an apparently regular gait. However, the fact that HR in ML was found to be significantly higher in overweight children seems to contradict, to some extent, this straightforward interpretation. This raises the need to rethink such concepts, or, at the very least, a specification of the way in which they should be interpreted depending on the considered context and source of possible gait anomalies. Here, we suggest that HR in the ML direction may be affected by specific biomechanical changes adopted by overweight children to optimize their walking efficiency (i.e., an increase in step width). However, this hypothesis needs further verifications, even considering that lateral trunk accelerations show different behavior with respect to those of AP in terms of variability. In any case, the use of HR, which relies on a relatively simple experimental approach and data processing, has certainly several advantages (limited intrusiveness, possibility to perform the tests under very ecological conditions), which makes its use recommendable as a screening tool to detect gait alterations early on that are associated with overweight and to assess the effectiveness of interventions targeted at improving mobility.

## Figures and Tables

**Table 1:** Demographic and anthropometric characteristics of participants. Values are expressed as mean ± SD.

	Normal Weight	Overweight
Participants # (M, F)	69 (32 M, 37 F)	39 (18 M, 21 F)
Age (years)	9.0 ± 1.3	9.0 ± 1.1
Height (cm)	132.3 ± 8.6	134.6 ± 8.9
Body Mass (kg)	28.1 ± 5.6	38.0 ± 6.7 ^†^
Body Mass Index (kg m^−2^)	15.9 ± 1.9	20.8 ± 1.5 ^†^

The symbol † denotes a significant difference vs. normal-weight children after Bonferroni correction (*p* = 0.0125).

**Table 2:** Spatio-temporal and smoothness-of-gait parameters calculated for the two groups of children. Values are expressed as mean ± SD.

		Normal Weight		Overweight	
		Absolute	Normalized	Absolute	Normalized
Spatio-temporal parameters of gait	Gait speed (m s^−1^ and s^−1^)	1.24 ± 0.21	0.97 ± 0.16	1.24 ± 0.28	0.96 ± 0.22
	Stride length (m and m m^−1^)	1.19 ± 0.18	0.92 ± 0.14	1.18 ± 0.21	0.92 ± 0.16
	Cadence (steps min^−1^ and steps min^−1^ m^−1^)	128.11 ± 13.84	99.71 ± 11.34	128.82 ± 12.36	99.89 ± 10.46
	Double support phase (% GC)	19.59 ± 3.16		18.98 ± 2.35	
Harmonic ratio	AP direction	95.13 ± 1.90		93.18 ± 4.75 ^†^	
	ML direction	80.35 ± 6.81		84.35 ± 8.65 ^†^	
	SI direction	94.09 ± 4.53		92.54 ± 6.72	

The symbol ^†^ denotes a significant difference vs. normal-weight children after Bonferroni correction (*p* = 0.0125 for spatio-temporal parameters, *p* = 0.016 for HRs): GC: gait cycle.

## Data Availability

The data presented in this study are available on request from the corresponding author.
